# Reducing vasospasm of vein and arterial conduits used in coronary artery bypass surgery: are solutions the solution or is preserved perivascular fat the answer?

**DOI:** 10.3389/fphys.2025.1539102

**Published:** 2025-01-31

**Authors:** Michael R. Dashwood, Zeynep Celik, Gokce Topal

**Affiliations:** ^1^ Surgical and Interventional Sciences, Royal Free Hospital Campus, University College Medical School, London, United Kingdom; ^2^ Department of Pharmacology, Istanbul University Faculty of Pharmacy, Istanbul, Türkiye

**Keywords:** perivascular adipose tissue, saphenous vein, internal thoracic artery, radial artery, vasospasm, antispastic solutions, coronary artery bypass surgery

## Abstract

The three main conduits used for myocardial revascularization in patients with coronary artery disease (CAD) are the internal thoracic artery (ITA), radial artery (RA) and saphenous vein (SV). In coronary artery bypass grafting (CABG) conduits may be harvested with perivascular adipose tissue (PVAT) intact (pedicled) or removed (skeletonized). Various studies have shown that the patency rate of these bypass grafts may be affected by the preservation or removal of PVAT. Vasospasm is often encountered at harvesting, a condition that has both immediate and long term effects on graft performance. During surgery a variety of antispastic solutions are routinely used on conduits that have anti-contractile and/or vasorelaxant actions. Spasm may be abolished or reduced when PVAT is left intact at harvesting and this is particularly the case for the SV. The protective properties of PVAT are multifactorial, ranging from its mechanical properties in supporting the graft after implantation to the beneficial effect of adipocyte-derived factors. This review aims to outline the possible mechanisms through which preserved PVAT could alleviate vasospasm and improve conduit performance in CABG. Moreover, since preservation of PVAT reduces spasm at and after surgery this review also considers whether antispastic solutions are needed if conduits are harvested with PVAT intact.

## Graphical Abstract

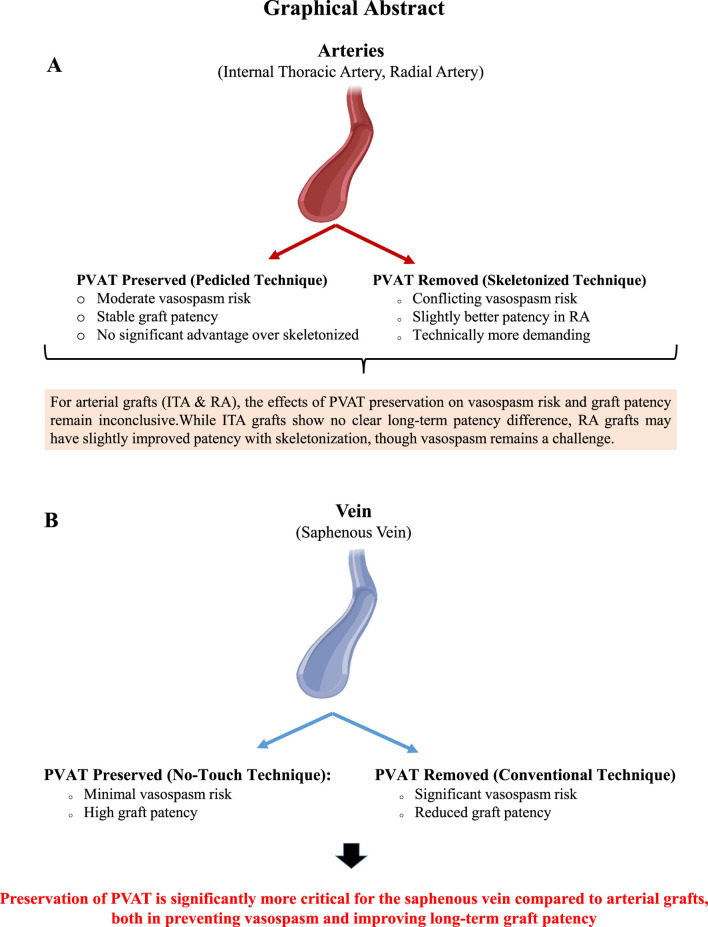

## 1 Introduction

**FIGURE 1:**
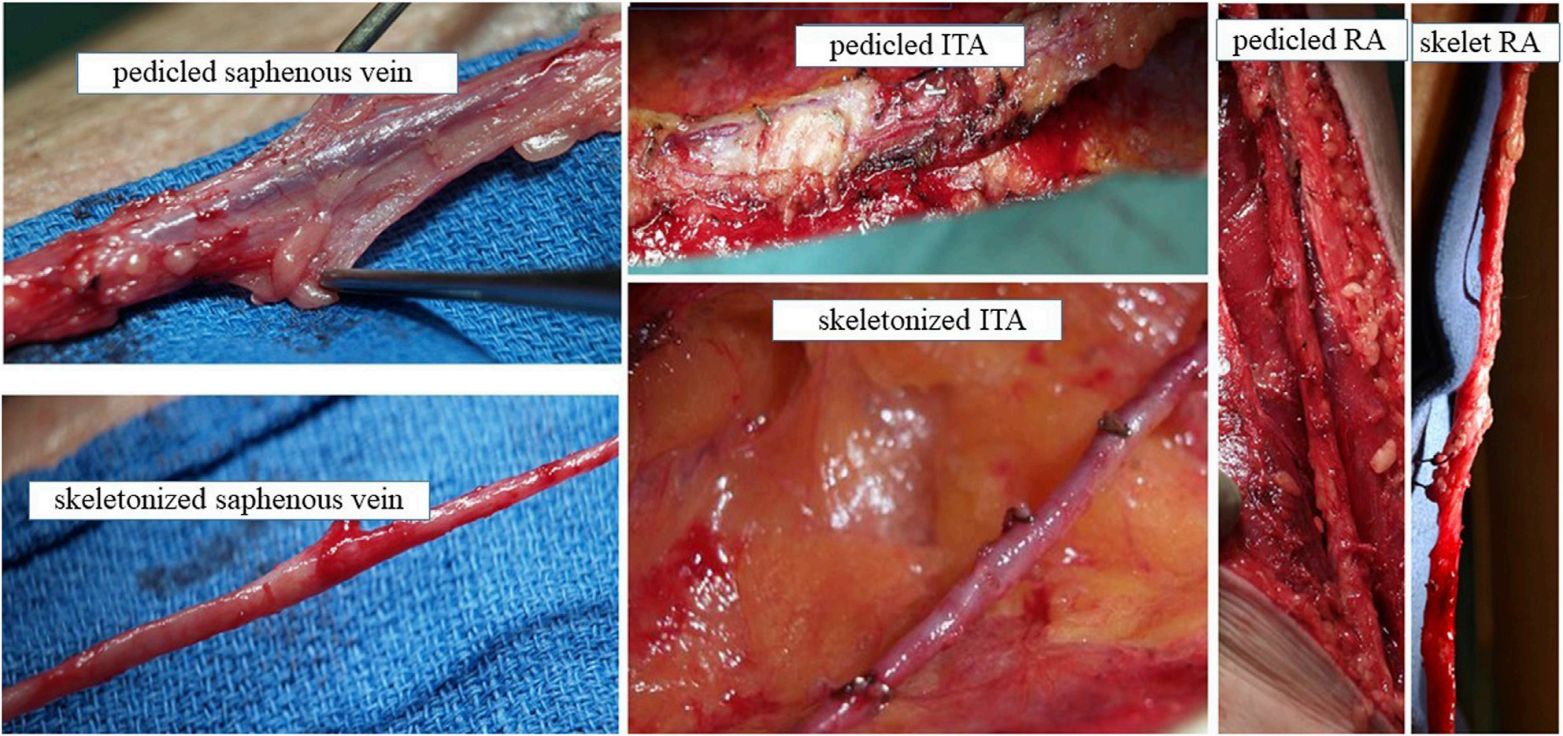
Conduit harvesting for CABG with PVAT intact and PVAT removed Explants of SV, ITA and RA with PVAT intact (pedicled) or with PVAT removed (skeletonized) at harvesting from patients undergoing CABG. We acknowledge Dr Domingos Souza, Orebro University Hospital, Sweden for providing these photographs.

**FIGURE 2:**
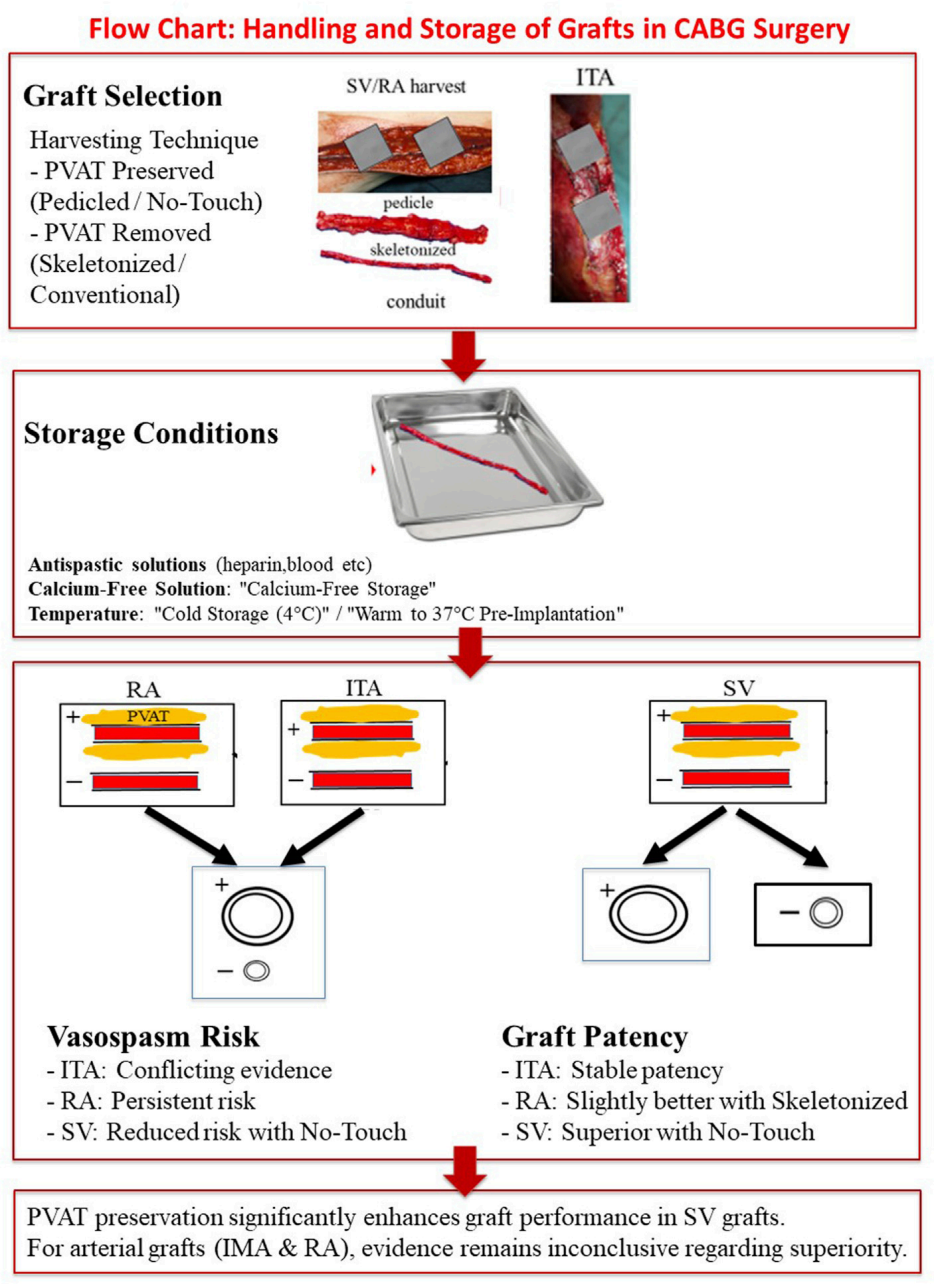
Flow chart: Handling and storage of grafts in CABG surgery.

Coronary artery disease (CAD) accounts for >7 million deaths per year worldwide with >800,000 coronary artery bypass graft (CABG) procedures performed annually (WHO 2021). The three main conduits used for CABG are the internal thoracic artery (ITA), radial artery (RA), and saphenous vein (SV) (Figure 1) (Dimeling et al., 2021). While the, *in situ,* ITA is recognised as the ‘gold standard’ graft (Yim et al., 2020; Alzghari et al., 2023) there are conflicting views as to whether the SV (Dreifaldt et al., 2013; Dreifaldt et al., 2021) or RA (Gaudino et al., 2018; Nappi et al., 2021) is the second graft of choice. Vasospasm occurs during harvesting in a high proportion of cases that is associated with surgical trauma and vascular damage that affects all vascular layers, the endothelium (Svendsen et al., 1986; Souza et al., 1999; Tsui et al., 2001), vascular smooth muscle cells (VSMC) (Ahmed et al., 2004; Verma et al., 2014), vascular nerves (Loesch and Dashwood, 2009; 2018; Thakore and Brain, 2017) and perivascular adipose tissue (PVAT) (Dashwood et al., 2011; Mikami et al., 2023; Sohn et al., 2024). Apart from the immediate effect during conduit harvesting when performing CABG, vasospasm may also have mid- and long-term adverse effects (Novaković et al., 2003; Gaudino et al., 2017). An early angiographic study proposed that spasm in a SV may have an important impact in the initial progression of myocardial infarction, graft occlusion, or recurrence of angina despite an originally successful CABG operation (Victor et al., 1981). Also, refractory vascular spasm, involving the entire coronary artery system and grafts following CABG, can be improved using early extracorporeal oxygenation and controlled cardiocirculatory assistance (Lorusso et al., 2012). A variety of antispastic strategies and/or drugs have been developed including submersion in heparinised blood, saline containing papaverine, nitroglycerine, nitric oxide (NO) donors and mixed anticontractile cocktails (Dashwood et al., 2024). Many experimental studies have investigated the relaxant/anticontractile effects of such treatments on both animal and human blood vessels. Of particular relevance to spasm is the use of *in vitro* organ bath studies aimed at determining the involvement of various cell types in controlling both relaxation and constriction of the vasculature. Indeed, such past experiments revealed the importance of endothelium-derived factors in vasomotor control (Kedzierski and Yanagisawa, 2001; Moncada and Higgs, 2006; Vanhoutte, 2018; Barton and Yanagisawa, 2019) and, more recently, the involvement of perivascular-derived factors (Ozen et al., 2015; Fernández-Alfonso et al., 2017; Cheng et al., 2018). When assessing vasoreactivity, *in vitro*, it is crucial to consider the potential involvement of such factors as overlooking their potential contribution may yield unreliable results (Dashwood et al., 2024). PVAT, which surrounds the blood vessels, plays a crucial role in maintaining vascular homeostasis. PVAT functions in both endocrine and paracrine roles by generating a wide variety of metabolically active biomolecules. The properties of PVAT implies that this outermost vessel layer plays a potentially significant role in reducing vasospasm and enhancing the efficiency of vascular conduits used in CABG. Preserved PVAT has been shown in several clinical investigations to support its function in improving the patency of conduits used in CABG. Vasospasm and graft occlusion were considerably less common in patients receiving artery grafts with intact PVAT than in patients receiving SV grafts without PVAT, according to a retrospective review of CABG patients (Mikami et al., 2023). In a similar manner, preservation of PVAT was linked to better graft patency and a reduced incidence of adverse cardiovascular events in a prospective randomized controlled trial comparing the results of pedicled *versus* skeletonized arterial grafts (Lamy et al., 2021). These results imply that protecting PVAT during graft preparation and harvesting can be a straightforward yet useful strategy to improve CABG performance (Ozen et al., 2013; 2015; Kociszewska et al., 2022). Even with this encouraging data, research on PVAT’s function in CABG is still ongoing. This review aims to give an overview of the mechanisms by which PVAT may protect vascular grafts during CABG. Furthermore, it examines the necessity of anti spastic solutions when vascular conduits are harvested with intact PVAT or with PVAT removed. To clarify the procedural steps, vessel handling is summarized in the accompanying flowchart (Figure 2).

## 2 Mechanisms of PVAT function

### 2.1 Release of bioactive molecules from PVAT

The blood vessel wall is comprised of three primary layers: intima layer is formed by endothelial cells (ECs); the media is predominantly composed of VSMCs; and adventitia is primarily constituted by fibroblasts (Miao and Li, 2012). There is no mechanical barrier between the vessel wall and the PVAT; rather, a fibrous layer separates adipocytes from adventitial cells. This arrangement facilitates direct communication among substances secreted by the PVAT and those released by the vessel wall (Zhang et al., 2021). The pivotal study by Soltis and Cassis (1991) first reported the anti-contractile effect of PVAT. Subsequently several studies have indicated that PVAT modulates many vascular functions that are essential for sustaining vascular homeostasis. PVAT releases a range of adipocytokines and chemokines (Soltis and Cassis, 1991). In physiological conditions, PVAT modulates vascular tone and reactivity through the release of several biologically active substances, including perivascular relaxing factors (PVRFs) and perivascular contractile factors (PVCFs) (Sigdel et al., 2024). The primary PVRFs include adiponectin, angiotensin (Ang) 1–7, leptin, omentin, NO, and hydrogen sulfide (H_2_S), while PVCFs mainly include superoxide and angiotensin II (Ang II) (Zhang et al., 2021; Rami et al., 2022) (Table 1). In another study, the anti-contractile effect of PVAT was determined to be independent of NO, cyclooxygenase pathways, and mediated by tyrosine kinase-dependent activation of ATP-sensitive K⁺ channels (Löhn et al., 2002).

**TABLE 1:** Role of PVAT-derived fact.

PVAT-derived factors	Effects	References
Adiponectin	Reduce inflammation	Hou et al. (2018) and Feij et al. (2020)
	Relaxation	Margaritis et al. (2013) and Tong et al. (2023)
Leptin	Relaxation	Bełtowski (2012), Dashwood and Tsui (2013), and Tong et al. (2023)
	Contraction	Rami et al. (2022)
Omentin	Reduce inflammation	Wang et al. (2020) and Zhou et al. (2020)
Nitric oxide	Reduce inflammation	Miao and Li (2012) and Tong et al. (2023)
	Relaxation	Saito et al. (2022) and Tong et al. (2023)
Hydrogen sulfide	Relaxation	Wang (2012) and Hillock-Watling and Gotlieb (2022)
Ang 1–7	Relaxation	Lee et al. (2009) and Wang (2012)
Angiotensin II (Ang II)	Contraction	Chang et al. (2020)
	Induce inflammation	Rami et al. (2022)
Chemerin	Contraction	Kotanidis and Antoniades (2021)
Prostanoids		
Prostacyclin	Relaxation	Chang et al. (2012)
Prostaglandins	Contraction	Ozen et al. (2013)
Resistin Serotonin (5-HT) Norepinephrine (NE) Monocyte chemoattractant protein-1 (MCP-1) Tumor necrosis factor-alpha (TNF-α) Interleukin-6 (IL-6)	Induce inflammation Contraction	Nosalski and Guzik (2017), Qi et al. (2018), Chang et al. (2020), Hillock-Watling and Gotlieb (2022), and Man et al. (2022)

A number of other adipocyte-derived anti-contractile factors have been identified in PVAT from arteries and veins used for CABG. Regional variations between arterial and venous adipocytes have been identified with those adjacent to the ITA being smaller than those in the PVAT of the SV (Shen et al., 2016). The recent study reported by Mikami et al. (2021) not only supports this finding but also showed that SV-PVAT and PVAT surrounding the ITA exhibited reduced fibrosis, diminished gene expression levels of fibrosis-related markers, and lower levels of metaflammation compared to PVAT surrounding the coronary artery and aorta. Also, this group describe elevated adiponectin gene expression level in the SV and ITA in agreement with localization and protein expression in these conduits as shown previously (Shen et al., 2016).

Adiponectin is among the most common adipokines released by PVAT and exerts both vasodilatory and anti-inflammatory effects on the vessel. Their action is mediated by two receptor types: adiponectin receptor 1 (AdipoR1) and adiponectin receptor 2 (AdipoR2) (Sowka and Dobrzyn, 2021). Adiponectin reduces inflammation by decreasing the expression of pro-inflammatory cytokines, including IL-6 and TNF-α, and by inhibiting the synthesis of cellular adhesion molecules through the suppression of the nuclear factor kappa-B (NF-κB) signaling pathway (Feij et al., 2020). In a study by Hou et al., adiponectin knockout mice exhibited increased expression of inflammatory markers, including TNF-α and MCP-1. Adiponectin promotes vasodilation by activating endothelial NO synthase (eNOS) through AMPK-mediated phosphorylation (Hou et al., 2018). This process results in increased NO production which is a powerful vasodilator (Tong et al., 2023). Also, adiponectin improves eNOS function by promoting phosphorylation and improving the synthesis of BH4, a critical cofactor necessary for eNOS activity (Margaritis et al., 2013).

Leptin is an adipokine that plays a crucial role in regulating appetite and body weight. Moreover, it is regarded as a protective adipokine with a beneficial impact on cardiovascular function (Tong et al., 2023). It is another PVAT-derived factor with potent vasorelaxant activity (Tong et al., 2023). Leptin-induced vasodilation occurs through specific mechanisms that are either endothelium-dependent or independent, contingent upon the specific type of blood vessel implicated. In large arteries, such as the aorta, leptin enhances endothelium-dependent vasodilation by activating AMP-activated protein kinase, a process comparable to that of adiponectin. This activation leads to the phosphorylation of eNOS, resulting in enhanced vasodilation. Additionally, leptin targets vascular ECs, inhibiting the contraction effects of Ang II by decreasing calcium release from cellular stores and stimulates the expansion of VSMCs (Bełtowski, 2012). Leptin has been localised in the PVAT of no touch SV using immunohistochemistry. Also, leptin protein was assessed in PVAT extracts by Western blot analysis and ELISA. The findings indicate that PVAT-derived leptin, as a potent vasodilator, may significantly contribute to the harvesting and enhanced long-term performance of no-touch SVs in patients undergoing CABG (Dashwood et al., 2011; Dashwood and Tsui, 2013; Fernández-Alfonso et al., 2017).

Omentin, also known as intelectin-1, and produced by PVAT, demonstrates anti-inflammatory effects by diminishing the expression of pro-inflammatory cytokines, including TNF-α, IL-6 and IL-1β, while augmenting the release of other anti-inflammatory adipocytokines, such as adiponectin and and IL- 10 through the inhibition of thioredoxin-interacting protein (TXNIP)/NLTP3 signaling pathway (Zhou et al., 2020). Also, omentin diminishes mitochondrial dysfunction, oxidative stress and the levels of pro-inflammatory cytokines such as, TNF-α, IL-6 and MCP-1, as well as cyclooxygenase-2 (COX-2) and prostaglandin E_2_ (PGE_2_) in macrophages induced by lipopolysaccharides (Wang et al., 2020). Various studies have indicated that omentin may exhibit a protective effect against endothelial dysfunction. In obese individuals, the established decrease of circulating omentin levels correlates with endothelial dysfunction (Çimen et al., 2017).

NO has a variety of important effects on blood vessels. In particular, NO released from ECs increases the diameter of the vessels by acting on VSMCs. Furthermore, NO positively affects the cardiovascular system by reducing inflammation and inhibiting oxidative stress in the vessel wall (Miao and Li, 2012). In the cardiovascular system, the effect of NO is mediated specifically through the enzyme endothelial nitric oxide synthase (eNOS) which is mainly expressed in the endothelium. This enzyme is expressed also in PVAT. eNOS-mediated NO has been demonstrated to show various anti-atherosclerotic effects, such as regulating VSMC proliferation, preventing leukocyte adhesion, inhibiting platelet aggregation, and reducing vascular inflammation (Tong et al., 2023). The vasodilatory effects of PVAT-mediated NO are produced by relaxing VSMCs via the cyclic guanosine monophosphate (cGMP) - protein kinase G (PKG) pathway and/or activating potassium channels in VSMCs to induce membrane hyperpolarization (Tong et al., 2023).

H_2_S is a gas produced by ECs, VSMCs and PVAT which is crucial in modulation of vascular tone. H₂S regulates vascular tone by preventing the proliferation of vascular cells and managing their autophagy and apoptosis (Hillock-Watling and Gotlieb, 2022). The vasodilatory effects of H_2_S occurs according to its capacity to activate large-conductance potassium (BK) channels in VSMCs. This activation results in hyperpolarization of the cytosol, afterwards leading to the inactivation of voltage-gated L-type calcium (Ca^2+^) channels. These conditions result in a decrease in intracellular Ca^2+^ concentration (Wang, 2012).

Ang 1-7, which is a part of the renin-angiotensin-aldosterone system, is known to be expressed in PVAT and promotes vasodilatation through interaction with the endothelium (Lee et al., 2009). Ang 1-7 activates eNOS via the endothelial Ang 1-7 receptor and enhances NO level. The augmentation in NO level results in vasodilatation via activation of BK channels (Wang, 2012).

### 2.2 Role of PVAT in vascular health and diseases

PVAT provides a supporting role that helps preserve vessel structure (Fernández-Alfonso et al., 2017). In a study by Greenstein et al., PVAT obtained from small arteries, isolated from subcutaneous gluteal fat biopsy samples of healthy individuals, was demonstrated to reduce the vasocontraction to norepinephrine (NE) when analyzed in a myograph system (Greenstein et al., 2009).

PVAT exhibits dysfunction in clinical situations including diabetes, metabolic syndrome and obesity. Its secretory profile changes are characterized by a diminished release of vasorelaxing factors, an increase of vasoconstricting factors and pro-inflammatory adipocytokines, including leptin, IL-6, TNF-α, and MCP-1**,** also infiltration of immune cells and proliferation of VSMCs (Rami et al., 2022; Sigdel et al., 2024). The accumulation of PVAT observed in obesity is believed to disrupt the balance between the secretion of injurious and beneficial adipokines released from PVAT (Ozen et al., 2015). These alterations lead to inflammation within PVAT, which unfavorably impacts on vascular function. PVAT can be damaged in various situations and becomes dysfunctional and has heightened the interest in the function of the outer layers of the vascular wall in the pathophysiology of vascular disease. Factors like diabetes, aging, obesity and mechanical stress can all lead to PVAT injury (Hillock-Watling and Gotlieb, 2022). These conditions collectively promote a state of elevated inflammation, which can result in the activation of PVAT cells, rendering them dysfunctional or pro-inflammatory. Dysfunctional PVAT is characterized by the secretion of various inflammatory and vasoconstrictive adipokines. Among these factors are reactive oxygen species (ROS), leptin, Ang II, serotonin (5-HT), NE, chemerin, resistin, MCP-1, TNF-α, and IL-6 (Qi et al., 2018; Chang et al., 2020). For example, the cytokine chemerin reduces NO availability by inhibiting the cofactor essential for eNOS, leading to its uncoupling and subsequent dysfunction (Kotanidis and Antoniades, 2021). The uncoupling of eNOS causes the production of ROS, thereby exacerbating vascular oxidative stress. IL-6, which is a pro-inflammatory cytokine secreted by PVAT, can directly influence ECs, resulting in elevated superoxide production and subsequent endothelial dysfunction (Nosalski and Guzik, 2017). Also, TNF-α involved in modulating the inflammatory response of PVAT, inhibits the expression of eNOS and promotes the production of ROS via the activation of the NF-κB signaling cascade (Nosalski and Guzik, 2017). Dysfunctional PVAT causes the elevation in recruitment of monocytes and T cells which produce even more inflammatory cytokines (Hillock-Watling and Gotlieb, 2022). Also, in this condition the cytokines which are generated to stimulate the proliferation of VSMCs, contribute to endothelial dysfunction, increase the secretion of pro-inflammatory cytokines, and inhibit the release of anti-inflammatory cytokines (Qi et al., 2018). Ang II, expressed in PVAT, causes inflammation by increasing the expression of adhesion molecules and cytokines, including MCP-1 and IL-6 (Rami et al., 2022). Also, Ang II has been reported to facilitate the infiltration of several immune cell types, such as T lymphocytes, M1 and M2 macrophages, and dendritic cells, into PVAT that causes the development of atherosclerosis and hypertension (Nosalski and Guzik, 2017). ROS generated by NADPH oxidase in PVAT contributes to endothelial dysfunction by depleting eNOS and altering perivascular inflammation (Rami et al., 2022).

## 3 Role of PVAT in coronary artery ByPass graft vessels

Various studies have investigated the role of PVAT in the vascular conduits that are used in CABG surgery. Table 2 summarises the effects of PVAT on coronary artery bypass grafts. In the following sections, the specific roles of PVAT will be discussed for each type of vessel used as a bypass conduit in CABG.

**TABLE 2:** Role of PVAT on CABGs *in vitro*.

CABGs	Study	Role of PVAT on CABG conduits	Potential mechanism
Internal thoracic artery (ITA)	Gao et al. (2005)	Vasorelaxation	Activation of Ca⁺^2^ -dependent K+ channels
	Ozen et al. (2013)	Vasorelaxation	Independently from prostanoids
	Kociszewska et al. (2015)	Vasorelaxation	Associated with adipocyte-derived relaxing factor
	Malinowski et al. (2008)	Vasorelaxation	Nitric oxide and prostacyclin-independent
	Malinowski et al. (2013)	Vasorelaxation	Involvement of Ca⁺^2^ dependent potassium channels
Radial Artery	Kociszewska et al. (2022)	Vasorelaxation	Associated with endothelial-independent adipocyte-derived relaxing factor secretion
Saphenous vein	Ozen et al. (2013)	Vasorelaxation	By releasing both PGE_2_ and prostacyclin (PGI_2_)
	Foudi et al. (2011)	Vasorelaxation	Through activation of EP4 and IP receptors
	Ford et al. (2006)	Vasorelaxation	Participation of L-type Ca⁺^2^ channels
	Kurazumi et al. (2022)	Improve patency	high-pressure intraluminal saline distension is avoided as spasm does not occur according to the no-touch technique
	Dashwood et al. (2007)	Improve patency	Associated with nitric oxide synthase activity
	Saito et al. (2022)	Source of NO	contributed to the eNOS protein content
	Souza et al. (2001) and Souza et al. (2006)	Reduce stenosis, improve patency	According to anti-spasmodic, anti-platelet, and anti-proliferative effects of PVAT

### 3.1 Radial artery

An *in vitro* study specifically investigated the vasorelaxing properties of PVAT of the human RA (Kociszewska et al., 2022). Here, isolated segments of skeletonized and pedicled RA were suspended in an organ bath and contracted to 5-HT to determine the concentration-effect relationship with and without PVAT. Skeletonized segments were precontracted with a single dose of 5-HT and 5-mL PVAT aliquots, from PVAT incubated in Krebs-Henseleit solution were transferred to the RA tissue bath inducing relaxation. Later, an effort was made to determine the involvement of ADRF in endothelium dependent vasorelaxation. Moreover, several potassium channel blockers were administred in order to investigate the role of potassium channels in the effect of ADRF. RA without PVAT exhibited more robust contraction in response to 5-HT than RA with PVAT. The PVAT aliquot induced relaxation in precontracted RA rings and ADRF is independent of endothelial vasorelaxants as evidenced by the unchanged vasorelaxant response following the addition of NG-monomethyl-l-arginine and indomethacin. These effects were also independent of potassium channel antagonists (Kociszewska et al., 2022).

### 3.2 Saphenous vein

SV graft patency is improved dramatically when the vein is harvested atraumatically and with its PVAT intact using the no-touch technique (Souza et al., 2001; 2006; Samano et al., 2015; Dreifaldt et al., 2021; Kurazumi et al., 2022; Ferrari et al., 2024). A study comparing no-touch with conventional SV grafts used immunohistochemistry to identify eNOS localization on vein graft sections and RT-PCR and Western blotting to assess eNOS mRNA and protein. NO synthase activity was measured using the citrulline assay (Dashwood et al., 2007). There was eNOS immunostaining of the endothelium of vein graft segments, with dense staining of the adipocytes and associated structures surrounding segments of no-touch SV. In addition, eNOS protein was identified in tissue extracts of both the vein and surrounding fat by Western blot analysis and NO synthase activity/NO generation confirmed using the citrulline assay. It was concluded that perivascular fat-derived NO plays a beneficial role in SVs harvested atraumatically and plays a role in the increased patency of these grafts in patients undergoing CABG. The study by Saito et al. (2022) also showed that the PVAT of SV is a source of NO. Here, NO levels of no-touch and conventional SVs were measured after 24 h of tissue culture. NO production was greater in no-touch compared with conventional SVs that was associated with PVAT. Interestingly, substantial levels of eNOS were expressed in PVAT (Saito et al., 2022) in agreement with the earlier study of Dashwood et al. (2007) who also demonstrated endothelium-dependent eNOS staining of the capillary network of the PVAT that is likely to have contributed to the total eNOS protein content of tissue extracts (Dashwood et al., 2007).

Based on results using no-touch SV it appears that PVAT plays a protective role and that its removal has a detrimental effect on graft patency (Dashwood and Tsui, 2013). It is not clear if the same is true for ITA or RA grafts since there is no clear evidence if patency rate is different whether the pedicle is intact or if the artery is skeletonized (Tatoulis, 2013; Kurazumi et al., 2022).

**FIGURE 3:**
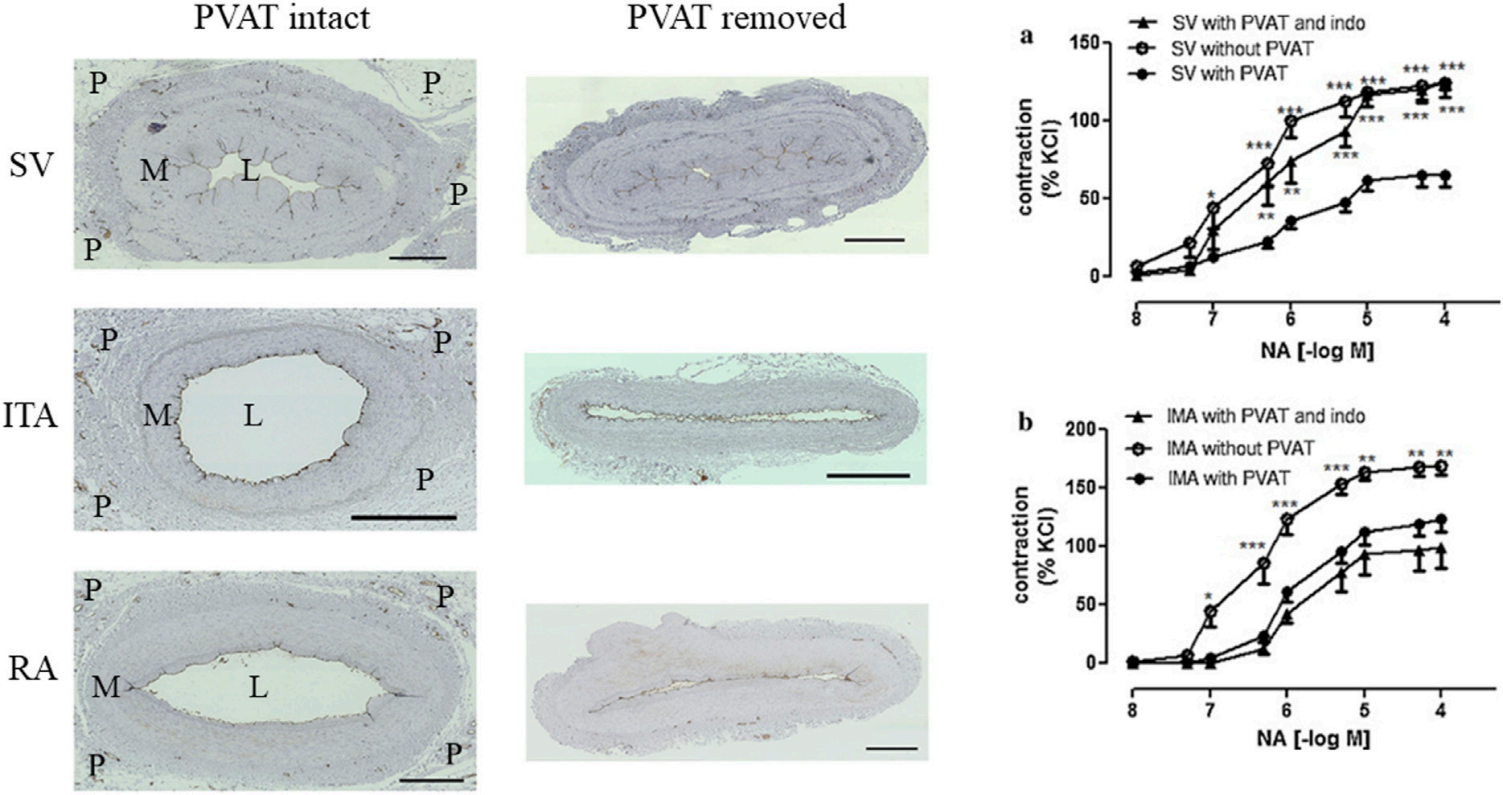
Left panel; Transverse sections of SV, ITA and RA with PVAT (P) intact and PVAT removed. The lumen (L) area is reduced in all conduits where PVAT is removed compared with those where PVAT remains intact. M = media. Adapted from unpublished data provided by Dr. Dashwood. Right panel; Anticontractile effects of PVAT on isolated SV and ITA conduits. Reproduced from Ozen et al. (2013) Elsevier. Reproduced with permission.

The surrounding PVAT may reduce stenosis of vein grafts by enhancing graft patency in patients undergoing CABG (Souza et al., 2001; 2006). This shows that PVAT plays an important role in preventing intimal hyperplasia and atherosclerosis following SV grafting (Souza et al., 2006). Various mechanisms have been proposed to explain the beneficial effects of PVAT in SV grafts, including anti-spasmodic, anti-platelet, and anti-proliferative effects, as well as mechanical properties that contribute to minimal damage to the vessel wall (Souza et al., 2001; 2006; Samano et al., 2021). Ford et al. and Ozen et al. have demonstrated that PVAT reduces vascular tone in response to noradrenaline through the involvement of L-type Ca⁺^2^ channels and by releasing both PGE_2_ and prostacyclin (PGI_2_) (Ford et al., 2006; Ozen et al., 2013). Studies conducted on the SV have indicated that the PVAT of the SV functions as a source of PGE_2_ and PGI_2_, which facilitate vasorelaxation of SV via EP4 and IP receptors, respectively (Foudi et al., 2011; Ozen et al., 2013) (Figure 3).

### 3.3 Internal thoracic artery (ITA)

Studies have suggested that maintaining the PVAT surrounding the ITA may be beneficial for preservation of graft function and structure by minimizing surgical trauma (Gao et al., 2005; Malinowski et al., 2008). Various researchers have demonstrated that the presence of PVAT diminishes vasoreactivity of ITA to phenylephrine/norepinephrine and U46619 (a thromboxane mimetic) (Gao et al., 2005; Ozen et al., 2013). The mechanism of vasorelaxation is thought to be dependent on the activation of Ca⁺^2^-dependent K^+^ channels but independent of prostonoids (Gao et al., 2005; Ozen et al., 2013) (Figure 3). Additionally, other studies confirmed that PVAT reduces the contractile response of the ITA to 5-HT and Ang II (Malinowski et al., 2008; 2013). It has been shown that the vascular tone reducing effects of PVAT are dependent on Ca⁺^2^-dependent potassium channels but independent of NO and PGI_2_ (Malinowski et al., 2008; 2013). Furthermore, Kociszewska et al. demonstrated that perivascular tissue reduces the contraction response of the ITA to phenylephrine but, interestingly, they discovered no association between the adipose tissue content and the degree of vasodilation of the ITA (Kociszewska et al., 2015).

## 4 Strategies for preventing of vasospasm in CABG conduits

Vasospasm is encountered when using the ITA, RA and SV at harvesting as conduits for CABG that causes problems not only during surgery but influences graft performance after implantation. Arterial grafts are more prone to spasm than the SV due to the relative absence of VSMCs in the media than in the ITA or RA. For some years the RA fell out of favour as a bypass conduit since it is exceptionally susceptible to vasospasm at harvesting that is of greater intensity and is often difficult to overcome, particularly during surgical manipulation at implantation of the graft. Indeed, revival of the RA as a conduit in CABG was mainly as a result of the successful use of antispastic treament (Acar et al., 1992). Apart from spasm that occurs at harvesting, spasm following CABG may be either a localised or a diffuse process leading to a reduced perfusion after disconnection of patients from cardiopulmonary bypass and observed in the intensive care unit (Jones et al., 1989). For arterial grafts, a classification system has been suggested where three types exist: Type I-somatic arteries; Type II-splanchnic arteries; and Type III-limb arteries. As a typical type I artery the ITA exhibits a higher endothelial function, releasing more NO and other relaxing factors than the RA. The RA, a type III artery, has a higher pharmacological reactivity to vasoconstrictors. Accordingly, the ITA has a superior long-term patency than the RA which requires a higher degree of pharmacological interventions to overcome the high incidence of spasm when using this vessel (He, 2013). Arterial spasm in CABG persists a clinical issue with refractory spasm occasionally resulting in fatal outcomes necessitating confirmation through angiography during and post-procedure (He and Taggart. 2016a). In a study on a large number of CABG patients, Lorusso et al. (2012) reported the clinical data and outcome where diffuse refractory vascular spasm was observed with electrocardiographic ischemic changes occurring within 8 h of surgery. Severe or refractory lethal spasm has also been reported as late as 15 h (Hosoba et al., 2012), 2 days (Harskamp et al., 2008) and extending from several months to years after harvest of these grafts (Suma, 1990; Mills et al., 1993; Hara et al., 1994; Cate et al., 1996).

### 4.1 Pharmacological approaches to vasospasm mitigation

#### 4.1.1 Nitrovasodilators

Nitrovasodilators, including glyceryl trinitrate (GTN), sodium nitroprusside (SNP), and isosorbide dinitrate, are commonly administered to patients undergoing CABG. These compounds act by releasing NO, a potent activator of guanylate cyclase and this enzymatic activation results in an elevation in cGMP levels within VSMCs, leading to a decline in intracellular Ca^2+^ concentrations causing VSMC relaxation (He and Taggart., 2016a). A previous study showed that GTN was more potent than SNP in its vasorelaxation effect, however SNP was more effective in inhibiting α-adrenoceptor and Ang II induced contraction in the ITA (He and Yang, 1997).

#### 4.1.2 Rho-kinase inhibitors

Perioperative spasm of the ITA and coronary arteries affects 0.43% of patients (Hou et al., 2023). Hou et al. evaluated the antispastic effect of a combination of the RhoA/Rho-kinase inhibitor, fasudil, with and without nitroglycerin in the ITA. Here, it was shown that fasudil fully relaxed certain vasoconstrictor-induced contractions and diminished protein level of ROCK2 in the ITA. It was also stated that when fasudil and nitroglycerin used together, this combination demonstrated a more pronounced impact than sole vasodilator (Hou et al., 2023). In another study, it has been reported that intraluminal fasudil injection was effective in suppressing spasm in the ITA of patients with ischemic heart disease who had undergone CABG (Watanabe et al., 2013).

#### 4.1.3 Calcium channel blockers (CCBs)

Calcium antagonists such as nifedipine, diltiazem and verapamil have been used alone or in combination with GTN (He and Yang, 1996b; He, 1998; Chanda et al., 2000) as antispastic drugs in arterial grafting. Ca^2+^ antagonists that are notably effective in preventing or curing K^+^-induced contraction in the ITA (He et al., 1989) or RA (He and Yang, 2000a) cause vasorelaxation of vessels by reducing Ca^2+^ influx by blocking voltage-gated (L-type) calcium channels. Diltiazem, although 15 times less effective than nifedipine in the ITA (He et al., 1989) and having little effect on human RA contractions (He and Yang, 2000b), facilitated the successful revitalization of the RA after this graft was neglected for about 20 years due to significant spasm issues (Acar et al., 1992). Two small randomized studies show no difference in graft patency between patients who used CCB and those who did not after RA grafting in CABG operations (Gaudino et al., 2001; Gaudino et al., 2005). Verapamil, which is available in injection form, has been shown to exhibit greater potency than diltiazem in human vasculature and therefore its inclusion in antispastic protocols has been recommended (Jett et al., 1988; He and Taggart, 2016b). In another study, the effect of a cocktail of nicardipine and nitroglycerin (30 μmol/L) on human ITA and RA was examined. The use of this cocktail was shown to provide a new antispastic procedure characterized by quick onset, complete vasorelaxation and superior prophylactic impact (He et al., 2008).

#### 4.1.4 Papaverine

Papaverine, an opioid derivative, causes vasodilation by inhibiting phosphodiesterase enzyme, which results in a reduction of Ca^2+^ influx and the inhibition of intracellular Ca^2+^ release (Pillai et al., 2020). Papaverine is not recommended for systemic use. There are also concerns about topical application of papaverine due to its acidic properties which may impair endothelial function (He and Taggart, 2016a). Mixing papaverine with blood for topical use (1% papaverine in intraluminal blood) is a commonly used method, which may prevent endothelial damage by reducing the acidity of papaverine through the buffering effect of blood. Studies have shown that the onset of the vasodilatory effect of papaverine is more gradual compared to other vasodilators (He and Yang, 1996b; He, 1998). In one study, the difference between the blood flow of ITAs administered topical papaverine only while as ITAs treated with a mixture of topical papaverine plus intraluminal papaverine, nitroglycerin and milrinone cocktail was observed and it was stated that treatment with the vasodilator cocktail provided a more effective increase in blood flow (Pillai et al., 2020).

#### 4.1.5 Phosphodiesterase inhibitors

Phosphodiesterases (PDE), classified into at least five types, play an important role in cardiac contraction and VSMC relaxation by hydrolyzing intracellular second messengers such as cyclic adenosine monophosphate (cAMP) and cGMP. PDE inhibitors are clinically important with their positive inotropic effects together with their vasodilator effects (He and Yang, 2000b). Milrinone is the PDE-III that increases intracellular cAMP, hence inhibiting myosin light chain kinase and causes vasodilatation in the vascular system (Pillai et al., 2020). Milrinone has been shown effective on the ITA (He and Yang, 1996a) or RA (He and Yang, 2000a) and it has been observed that the effect of milrinone on the ITA is more potent than its effect on the RA (Yamaguchi et al., 2006).

### 4.2 Effect of PVAT preservation on vasospasm

Endothelial dysfunction plays a major role in spasm after CABG that may be due to vascular trauma at harvesting. In this regard, PVAT may be seen as a mechanical ‘protector’, particularly for the SV (Tsui and Dashwood, 2002). Apart from an involvement of the endothelium, other mechanisms involved in spasm include mechanical factors (Dashwood, 2019; Samano et al., 2021), an effect on vascular nerves (Loesch and Dashwood, 2018; Saxton et al., 2018), PVAT and related adipocyte-derived factors (Fernández-Alfonso et al., 2017).

Of the three main conduits used in CABG, the ITA has the best patency and is accepted as the ‘gold standard’ graft. Although the SV is the most commonly used conduit in CABG there is some disagreement as to whether the RA or SV is the second graft of choice. Historically, arterial grafts were originally harvested with pedicle of PVAT intact and the SV with PVAT removed. Over the last decades this has changed with all conduits prepared as either pedicled or skeletonized grafts with varying opinions as to which preparation is best at reducing spasm and improving graft patency. The ITA, RA, and SV are traditionally harvested through a long incision and blunt dissection, with alternatives used, e.g., electrocautery or harmonic scalpel. RA and SV are fully excised as free grafts, while the ITA remains *in situ*. Pedicled grafts, prepared with intact PVAT, maintain normal vessel structure and avoid the need for distension, particularly in the SV. In contrast, skeletonization removes PVAT, causing vascular damage, including endothelial denudation, media thinning, and adventitial injury, and may induce spasm, especially in the SV (Souza et al., 2006) (Figure 3). In the following section the methods of removing PVAT and its effect on graft performance are described.

#### 4.2.1 Internal thoracic artery

Free flow in ITA grafts has been compared between those harvested with PVAT intact (pedicled) and those with PVAT removed (skeletonized) in patients undergoing CABG. Here the internally versus externally applied low dose papaverine effects on free flow from distal ITA grafts were compared, initially at the early stage of cardiopulmonary bypass and secondly just before completion of grafting. The first flow, with external application of papaverine alone, was superior in the skeletonized ITA grafts compared to pedicled ITA grafts (38.9 ± 15.8 vs. 18.0 ± 6.8 mL/min; p < 0.001). The second flow showed the pedicle grafts with intraluminal papaverine injection and the skeletonized ITAs exhibited superior flow compared to pedicled grafts with external papaverine application (67.4 ± 25.5 and 59.7 ± 22.5 vs. 38.1 ± 13.1 mL/min; p < 0.005 and p < 0.05, respectively). It was concluded 1) that skeletonization of the ITA is as efficient at increasing free flow as internal papaverine application for the pedicled graft and, 2) that spasm in skeletonized ITA can be reduced and that reduced early flow can be avoided without internally applied papaverine (Choi and Lee, 1996). In a later study, the effects of vascular damage due to skeletonization of the ITA was assessed. It was concluded that skeletonization of ITA compared with an intact pedicle in CABG was not associated with an increased risk of complications or elevated mortality in the early period following surgery (Belov et al., 2005)**.** The effect of dilators on graft flow has been studied in a porcine ITA graft model using a computer-controlled perfusion system. Here, the effects of increasing doses of NE was measured under constant inflow pressure and fixed outflow resistance. Under these conditions the effects of extraluminal or intraluminal nitroglycerin, nitroprusside, verapamil or papaverine were assessed at fixed time intervals (baseline, immediate and 2 h). Arteries treated with verapamil and papaverine had a longer duration of efficacy in preventing vasospasm compared to those treated with nitroglycerin and nitroprusside. Extraluminal administration of papaverine in the ITA was suggested to be most effective, likely because of the extended exposure provided by this route of administration. Interestingly, it was shown that the effects of verapamil and papaverine were more prolonged in the gastroepiploic artery (a conduit now very rarely used) when administered extraluminally and suggested to be potentially attributable to absorption in the PVAT and subsequent gradual release (Montgomery et al., 1996).

The results from a large observational cohort study show significantly increased rates of graft occlusion (9.6% vs. 3.9%; adjusted odds ratio: 2.41; P = 0.002), major adverse cardiovascular events (7.1% vs. 2.1%; adjusted hazard ratio: 3.19; P = 0.002), and repeat revascularization (5.0% vs. 1.4%; adjusted hazard ratio: 2.75; P = 0.03) in skeletonized ITA grafts compared to pedicled grafts (Lamy et al., 2021). However, another study suggested no significant differences between skeletonized and pedicled ITA techniques indicating moderate-level evidence that neither technique offers a clear advantage in mid-term outcomes (Sun et al., 2015).

#### 4.2.2 Radial artery

A single centre 1 year follow up study on skeletonized RA grafts used in CABG reported that excellent 1-year patency rates for skeletonized RA grafts, with a graft patency rate of 95.2% and an anastomosis patency rate of 97.2% but that careful examinations of RA grafts are required in patients with a history of peripheral artery disease and diabetes (Hirose and Amano, 2004)**.** Another study on the RA used in CABG compared conventional RA grafts, with pedicle intact, with grafts harvested with a long posterior fasciotomy, a similar procedure to skeletonization. Blood flow index was measured in both groups, comparing pedicled with skeletonized RAs before and after fasciotomy. Flow in patients where skeletonized RA had been used increased significantly when compared with pedicled RA. It was concluded that RA skeletonization aimed to reduce spasm should not be the only option at harvesting suggesting that subfacial RA dissection provides an increased blood flow index in addition to a reduction in manipulation time and vascular injury of the graft (Kucukarslan et al., 2008)**.** The study by Rukosujew et al. describes a surgical approach comparing pedicled or skeletonized RA. Here, the RA was prepared ‘conventionally’ using scissors with pedicled or skeletonized RA prepared using an ultrasonic scalpel (Rukosujew et al., 2004). Papaverine was used in all cases to prevent spasm during and after harvesting. Tissue specimens from each group were used for the analysis of endothelial integrity by scanning electron microscopy and blood flow measured after implantation of RA in all groups with a flow probe. Mean blood flow through the graft after completion of surgery was similar among all groups. However, skeletonization utilizing scissors and clips was more time consuming and technically challenging although produced considerably longer grafts. Notably, skeletonization with an ultrasonic scalpel was more commonly linked to severe endothelial damage whereas pedicled preparation utilizing scissors or an ultrasonic scalpel was much simpler and faster, without compromising endothelial integrity.

#### 4.2.3 Saphenous vein

CABG surgery continues to be the conventional intervention for multivessel CAD with the SV remaining the most commonly used conduit of choice in approximately 80% of all cases worldwide (Mack et al., 2021). The SV was first proposed as a graft by Favaloro (Favaloro, 1969) who emphasized that, “Care must be taken to dissect only the vein, avoiding as much as possible the adventitia that surrounds it”. The SV has several benefits as its greater length and ease of access provides more graft material compared to arterial conduits (Tsui and Dashwood, 2002). However, preparing the SV in this manner, the cushion of surrounding fat is removed, and the adventitia is damaged. Paradoxically, while arterial grafts are most commonly harvested with their outer pedicle intact, according to Favaloro’s original instructions the SV pedicle should be removed. Before grafting, an average vein experiences low pressures (∼5–8 mmHg), nonpulsatile flow, and a shear stress of around 0.2 dyne/cm^2^. However, following transplantation into the arterial system the vein will experience elevated pressures (∼60–140 mmHg), pulsatile flow, and a shear stress of around 3–6 dyne/cm^2^ (Lemson et al., 2000). This exposure of the SV to arterial haemodynamics is associated with many features of vein graft occlusion (Jeremy et al., 2007; Fan et al., 2017). SV graft patency is improved dramatically when the vein is harvested atraumatically and with its PVAT intact using the no-touch technique (Samano et al., 2015; Tian et al., 2021; Ferrari et al., 2024). Results from a 16-year follow-up study provided strong evidence demonstrating the superiority of no-touch SV grafts over conventional grafts, with patency rates comparable to IMA (83% vs. 88%) (Samano et al., 2015). Moreover, the randomized controlled trial by Dreifaldt et al. (2021) demonstrated that no-touch SV grafts have superior coronary anastomosis patency rates (91% vs. 81%, P = 0.046) and perform better in moderate stenosis (70%–89%) scenarios (95% vs. 74%, P = 0.017), providing evidence supporting their use as a preferred conduit in specific clinical situations (Dreifaldt et al., 2021). In addition, another study provided evidence emonstrating that no-touch -SV grafts exhibit superior long-term patency (83% at 16 years, P = 0.03) compared to conventional ones (Mikami et al., 2024). When using the no touch technique high pressure intraluminal saline distension is obviated since spasm does not occur as direct handling of the vein by surgical instruments is avoided. Also, the surrounding cushion of PVAT provides mechanical support to the vein and is also a source of adipocyte-derived factors beneficial to graft performance (Dashwood et al., 2011; Fernández-Alfonso et al., 2017; Mikami et al., 2024). Based on results using no-touch SV it appears that PVAT plays a protective role and that its removal has a detrimental effect on graft patency (Dashwood and Tsui, 2013).

## 5 Using distension for prevention of vasospam

**FIGURE 4:**
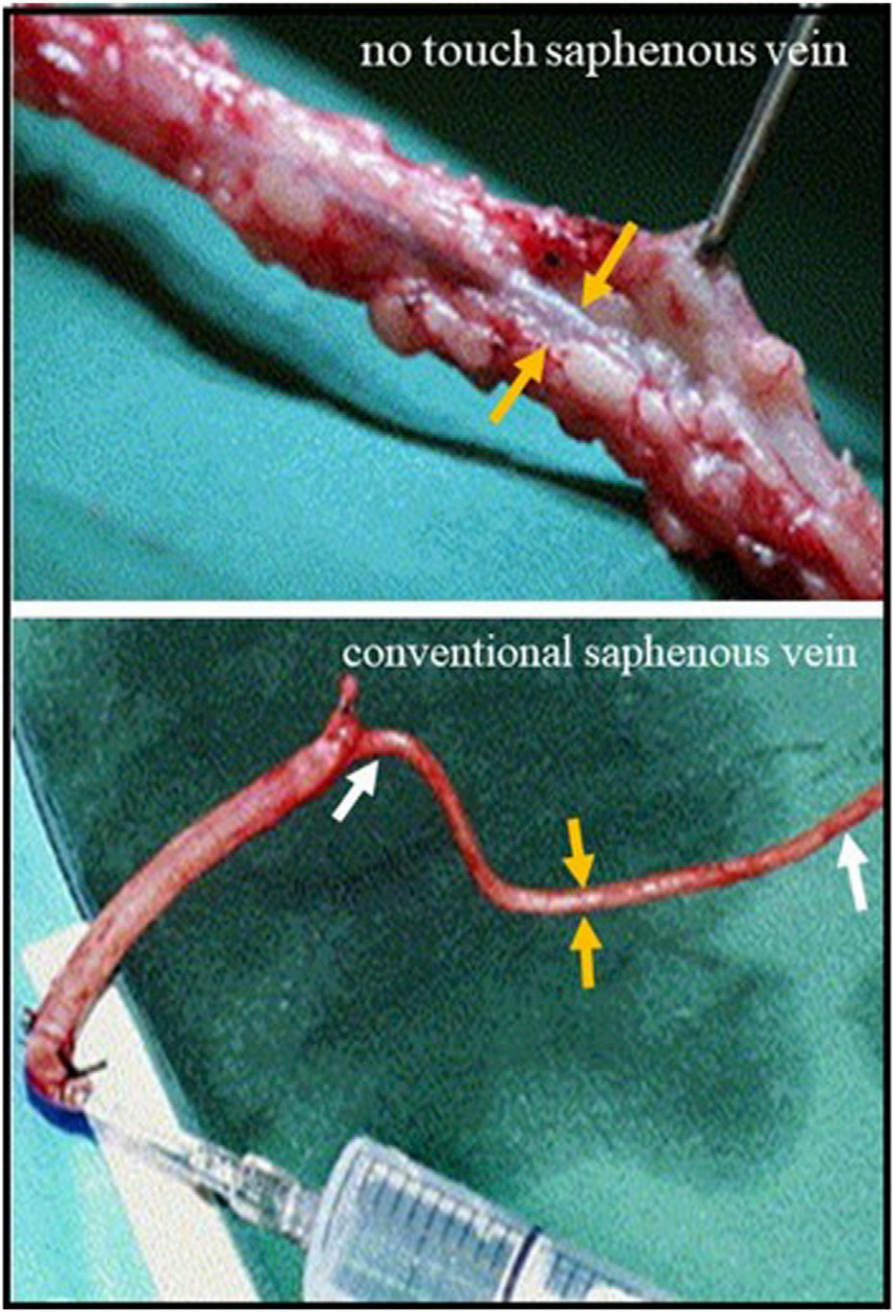
Saphenous vein harvesting using conventional and no touch techniques Top panel; No touch saphenous vein with surrounding PVAT intact. The orange arrows (correct) show the vein which does not go in to spasm. Lower panel; Conventional saphenous vein with PVAT removed. The length of vein between the white arrows has gone into spasm that is overcome using high pressure intraluminal saline distension through a syringe. The orange arrows show the reduced vein thickness.

It is interesting to note that, while the arterial grafts had for many years traditionally been harvested with PVAT preserved, the SV was harvested with PVAT removed using the conventional technique described by Favaloro (Favaloro, 1969). As previously mentioned, the SV is the conduit of choice due to its superficial position, long length and ease of access (Dashwood and Tsui, 2013). However, until recently it was accepted that the patency of the SV was inferior to the ITA where SV graft failure occurs in 50% of cases within 10 years after surgery compared with about 5% for the ITA over the same period (Jeremy et al., 2007). Like the ITA and RA spasm occurs at SV harvesting with a number of pharmacological strategies used to reduce this problem, not only during CABG but post-operation using a variety of anti spastic agents such as GTN, papaverine hydrochloride and verapamil (He et al., 1993). Apart from the use of vasodilators, ‘manual’ intraluminal distension is used when harvesting the SV to overcome spasm (Figure 4). This is an important issue as venous pressure *in vivo* is normally ∼10 mmHg whereas the SV graft is subjected to an elevated coronary artery pressure of >100 mm Hg at completion of CABG. Galea et al. (1999) measured controlled intraluminal pressure using a manometer at pressures of 350–400 mmHg showing that surgical preparation of SV at harvesting increases expression of c-fos mRNA and apoptosis compared with non-distended SV, concluding that pressure changes may influence the subsequent failure rate of SV grafts. In fact, the study by Roubos et al. (1995) suggests that the average peak distension pressure required to overcome SV spasm at harvesting was 480 mmHg, a pressure that inflicts damage to all layers of the vessel wall (Stigler et al., 2012). The atraumatic, no-touch, technique introduced by Souza, (1996) harvests the SV with PVAT intact. Here, spasm is almost completely abolished, and the use of antispastic solutions is unnecessary. By leaving PVAT intact, direct handling of the SV by surgical instruments is avoided and spasm does not occur. By preserving SV PVAT vascular damage is reduced and graft patency improved dramatically when compared with conventional, skeletonized SV grafts and to a rate comparable to the ITA (Souza et al., 2006; Samano et al., 2020). The protective role of SV PVAT is partly mechanical, as it protects the endothelium, VSMCs and vasa vasorum from the effects of 300 mmHg intraluminal pressure (Dashwood et al., 2009; Samano et al., 2020) (i.e., ∼3 times coronary artery pressure). Also PVAT is a source of anticontractile factors (Fernández-Alfonso et al., 2017). Thus, the removal of PVAT and use of hight pressure distension can result in both an immediate and long term reduction in graft patency. Therefore, at harvesting, the risk of distension-induced endothelial and vascular damage effects needs to be considered.

An early study by He et al., noted the spasm that occurs at harvesting the SV for CABG and the high pressure distension required to overcome this problem (He et al., 1993). Since the high pressures used damage the intima and media, promoting vein graft occlusion, the authors studied the relaxant effect of various vasodilators on human SV grafts *in vitro*. Organ baths studies were performed on human SV segments precontracted with KCl or a thromboxane mimetic and then the effects of vasodilator agents assessed. GTN or papaverine hydrochloride resulted in 80%–100% relaxation of contraction elicited by KCl or thromboxane. Verapamil completely abolished KCl-induced contraction and reduced the thromboxane contraction by 75%. Time course investigations indicated that GTN exhibited a quick onset and short duration of peak effect, whereas, similar to papaverine, verapamil demonstrated delayed onset and prolonged duration. A combination of GTN and verapamil resulted in rapid onset with long duration of action and this mixture subsequently used and applied both topically and intraluminally during SV harvesting for CABG. In their extensive review Rosenfeldt and colleagues provide an excellent overview of the pharmacology of coronary artery bypass grafts (Rosenfeldt et al., 1999). Moroever, they mention that the pressure required to overcome spasm (>400 mmHg) during graft harvesting can cause endothelial and vascular damage and can result in immediate and long term reduced graft patency.

## 6 Conclusion

Despite there being a number of studies on PVAT and its effects on conduits used in CABG surgery, certain challenges exist. Up to date, for the ITA and RA, the role of PVAT/ADRFs in preventing spasm is unclear. While for many years spasm in these conduits was overcome using a variety of vasodilators, more recently the removal of PVAT has been shown to also have antispastic effects on these conduits used for CABG. This suggests that PVAT may have no, or little anti-contractile action in these arterial grafts in patients undergoing CABG. This finding in arterial grafts presents a conundrum since there is experimental evidence that PVAT has an anticontractile effect on these vessels in *in vitro* studies. The situation seems more clear for the SV that has been treated with a variety of vasodilators to overcome spasm encountered using the conventional harvesting technique where PVAT is removed. Whereas this spasm has previously been overcome using a combination of dilators and intraluminal high pressure distension, it is now recognised that spasm is avoided when the SV PVAT is left intact, a situation that has been confirmed *in vitro*. Discrepancy between PVATs role in arterial grafts and SV can be explained by other factors that are involved in the spasm of these vessels, such as denervation, endothelial and/or general vascular damage. On the other hand, *in vitro* studies using isolated vessels may not accurately represent *in vivo* conditions. Too often there is a lack of information regarding the endothelial status of the vessels used in experimental studies. In fact, it is essential to assess both endothelium-dependent and independent vasorelaxation in *in vitro* ring preparations of conduits used in CABG. Furthermore, diversity in patient population and graft types complicates exploitation of the results. This review provides an overview of the role of PVAT and the possible mechanisms through which preserved PVAT could alleviate vasospasm and improve conduit performance in CABG surgery. Additional randomized controlled trials to compare PVAT-preserved versus skeletonized grafts are required to confirm the results of earlier research and to refine the techniques for preserving PVAT in surgical applications. In conclusion, the therapeutic advantages of PVAT preservation in CABG offers a possible path forward for enhancing surgical results. However, its incorporation into clinical practice and guidelines needs furher validation.
